# The hidden burden of adult allergic rhinitis: UK healthcare resource utilisation survey

**DOI:** 10.1186/s13601-015-0083-6

**Published:** 2015-11-18

**Authors:** David Price, Glenis Scadding, Dermot Ryan, Claus Bachert, G. Walter Canonica, Joaquim Mullol, Ludger Klimek, Richard Pitman, Sarah Acaster, Ruth Murray, Jean Bousquet

**Affiliations:** University of Aberdeen, Aberdeen, UK; The Royal National Throat, Nose and Ear Hospital, London, UK; Woodbrook Medical Centre, Loughborough, UK; University of Edinburgh, Edinburgh, UK; Upper Airways Research Laboratory, Ghent University Hospital, Ghent, Belgium; Allergy and Respiratory Clinic, IRCCS AOU S. Martino, Genoa, Italy; Hospital Clínic, IDIBAPS, CIBERES, Barcelona, Catalonia Spain; Center for Rhinology and Allergology, Wiesbaden, Germany; ICON, Oxford, UK; Acaster Consulting, London, UK; Medscript Ltd, Dundalk, Ireland; University Hospital, Montpellier, France; MACVIA-LR, Contre les Maladies Chronique spour un Vieillissement Actif en Languedoc Roussilon, European Innovation Partnership on Active and Healthy Ageing Reference Site, Montpellier, France; INSERM, VIMA : Ageing and Chronic Diseases, Epidemiological and Public Health Approaches, U1168, Paris, France; UVSQ, UMR-S 1168, Université Versailles St-Quentin-en-Yvelines, Versailles, France

**Keywords:** Allergic rhinitis, UK, Symptom episode, Co-medication, Absenteeism, Presenteeism

## Abstract

**Background:**

The affliction of allergic rhinitis (AR) has been trivialised in the past. Recent initiatives by the European Academy of Allergy & Clinical Immunology and by the EU parliament seek to rectify that situation. The aim of this study was to provide a comprehensive picture of the burden and unmet need of AR patients.

**Methods:**

This was a cross-sectional, online, questionnaire-based study (June–July 2011) including symptomatic seasonal AR (SAR) patients (≥18 years) from a panel. SAR episode pattern, severity, medication/co-medication usage, residual symptoms on treatment, number of healthcare visits, absenteeism and presenteeism were collected.

**Results:**

One thousand patients were recruited (mild: n = 254; moderate/severe: n = 746). Patients with moderate/severe disease had significantly more symptomatic episodes/year (8.0 vs 6.0/year; p = 0.025) with longer episode-duration (12.5 vs 9.8 days; p = 0.0041) and more commonly used ≥2 AR therapies (70.5 vs 56.1 %; OR 1.87; p = 0.0001), looking for better and faster nasal and ocular symptom relief. The reported symptom burden was high irrespective of treatment, and significantly (p < 0.0001) higher in the moderate/severe group. Patients with moderate/severe AR were more likely to visit their GP (1.61 vs 1.19 times/year; OR: 1.49; p = 0.0061); due to dissatisfaction with therapy in 35.4 % of cases. Patients reported SAR-related absenteeism from work on 4.1 days/year (total cost to UK: £1.25 billion/year) and noted presenteeism for a mean of 37.7 days/year (vs 21.0 days/year; OR 1.71; p = 0.0048). Asthma co-morbid patients reported the need to increase their reliever- (1 in 2 patients) and controller-medication (1 in 5 patients) if they did not take their rhinitis medication.

**Conclusions:**

This study differentiated between patients with mild and moderate/severe AR, demonstrating a burden of poorly controlled symptoms and high co-medication use. The deficiency in obtaining symptom control with what are currently considered firstline treatments suggests the need for a novel therapeutic approach.

**Electronic supplementary material:**

The online version of this article (doi:10.1186/s13601-015-0083-6) contains supplementary material, which is available to authorized users.

## Background

Allergic rhinitis (AR) has been trivialized over the years, despite its prevalence, chronicity and the burden it imposes on individuals and society [1–7]. Fortunately, the burden of AR is now being recognised both by the European Academy of Allergy & Clinical Immunology (EAACI) as well as at the EU parliament level, in order to highlight the profound impact this prevalent condition has on the quality of life (QoL) of AR sufferers and their families [8, 9]. Furthermore, the Polish presidency of the EU has highlighted the importance of early diagnosis and management of allergic diseases to promote active and healthy ageing [10], and made this an EU priority [11, 12]. All of these initiatives represent a fundamental shift in the perception of AR.

Reports in the literature already tell us that the daily burden of AR symptoms can be intrusive and debilitating, negatively impacting patients’ QoL [4, 5], normal activities [6, 13], well-being, cognitive functioning [14] even mood [15] and sleep [16]. Most AR patients attending their healthcare provider have persistent disease, with many using multiple therapies [17]. AR imposes a high socioeconomic burden, particularly in terms of indirect costs, including absenteeism and presenteeism (i.e. productivity loss or under-performance at work and school) [18–21]. It has also been associated with poor asthma control; patients reporting severe rhinitis exhibit poorer asthma control than those with mild disease, with a negative impact equivalent to that of smoking [22].

Most AR patients visiting their physician have moderate/severe disease with persistent symptoms [2, 17, 23–25]. Insufficient symptom control by currently considered firstline therapies has been identified as a major concern [2, 4, 17], a situation which has not improved over time [6, 7]. Co-medication is common; patients self-medicate and doctors co-prescribe (anti-histamines and intransal corticosteroids (INS) predominantly) [2, 3, 23, 26, 27] despite lack of evidence for this strategy in the literature [28–30]. AR patients have high expectations from their treatment [31], but most are dissatisfied with the results [32, 33]. Up to 40 % of patients have residual moderate/severe symptoms even after specialized treatment [17]. Management is often complicated by polysensitization [13, 34], the presence of allergic and non-allergic disease in the same patient (i.e. mixed rhinitis) [35] and confounded by phenotypes such as severe chronic upper airway disease (SCUAD) [36].

Clinical trials assess patients with the most severe symptoms with insufficient information from observational studies to understand the differences in burden between mild and moderate/severe rhinitis. To date, many surveys on the burden of AR have been conducted in Europe [2–5, 25] and in the US [6, 23, 37] but no cross-sectional questionnaire-based study, has assessed seasonal AR (SAR) episode pattern and duration, medication and co-medication usage (and the reasons for co-medicating), characterized residual symptoms on treatment nor provided information on healthcare visits, impact on asthma medication usage, absenteeism and presenteeism in a single study, stratified by disease severity (i.e. mild and moderate/severe).

The aim of this study was to describe the burden and unmet need of AR in one study, stratifed by disease severity. AR patients have been included in hundreds of clinical trials without a true understanding of the real burden of this disease, the way patients experience their symptoms and how they and their health care provider manage their disease in real-life. A secondary aim was to use the data obtained to inform future AR clinical trial design and result relevancy.

## Methods

### Study design

This was a cross-sectional, online, questionnaire-based study designed to collect representative views of people diagnosed with SAR. It was carried out in the UK between June and July 2011. The survey content was informed by experts (see Additional file 1). Experts contributed to all aspects of the survey from item and response level development and provision of key concepts to explore to provision of full UK AR medication listings. Ethics approval was obtained from Independent Investigational Review Board Inc., (Florida, USA). Concept elicitation interviews with five patients were conducted prior to the start of the study to establish the most effective way to capture data with the least patient burden. These interviews were designed to ensure patient comprehension of the questions asked. Additional information to describe terms included in the survey were included based on patient advice.

### Recruitment, patients and data collection

Potential participants from a UK patient panel database (Opinion Health) were contacted about taking part in the study. This is an extensive database of patients with a variety of medical conditions, who gave prior consent to be contacted for research purposes. Patients are recruited into the Opinion Health panel from various channels, including direct mailing, bespoke telephone recruitment, peer/healthcare provider referral, magazine/newspaper advertising, and from relevant charities/associations/communities. The wide range of recruitment methods employed has led to a strong and nationally representative sample of the general population of which 18 % are aged over 65 years (30 % who are 55+ years), over 35 % are from lower household income bands with 17 % from Social Grade D or E.

These potential participants were provided with the survey address and unique identifier, which they could use to access the online survey. Participants who followed the link were presented with a study screening form to assess their eligibility. Patients (≥18 years of age), currently residing in the UK, with a self-reported clinical diagnosis by a medical professional of SAR and currently experiencing rhinitis symptoms, were recruited after informed consent. Currently symptomatic patients were selected to minimize recall error, enabling patients to draw on current symptomatic experience. Patients who experienced AR symptoms all year round (i.e. perennial allergic rhinitis) with no seasonal flare-ups were excluded.

The survey was sent to 1300 potential participants. The aim was to recruit 1000 SAR participants, 200 mild and 800 moderate/severe. For the purpose of screening, disease severity was graded using the ARIA-defined criteria of sleep disturbance, impairment of daily activities including leisure/sports, impairment of work/study and presence of troublesome symptoms [1].

### Surveys

All eligible participants were granted online access to the main survey to be completed at their own pace. Patients next completed symptom severity and socio-demographic/healthcare utilisation questionnaires (see Additional file 1). Symptom severity was assessed by EMA and FDA endorsed efficacy endpoints 12 h reflective total nasal symptom score (rTNSS; consisting of nasal congestion, itching, rhinorrhea and sneezing) and 12 h reflective total ocular symptom score (rTOSS; comprising ocular itch, redness and watering). These reflective scores assess symptom severity for the previous 12 h. Patients rated all symptoms as ‘none = 0’, ‘mild = 1’, ‘moderate = 2’ or ‘severe = 3’, both for symptoms ‘today’ and for symptoms ‘at their worst’. Socio-demographic Information collected included patients’ age, gender, ethnicity and educational level. The healthcare resource utilisation survey included questions on duration and number of SAR symptom episodes, SAR medication usage, GP visits, impact on co-morbid asthma, absenteeism and presenteeism. These latter two items were based on the Work Productivity and Activity Impairment (WPAI) questionnaire. The full WPAI questionnaire was not used in order to minimise participant burden. Symptom episode was defined for patients as ‘an episode is a period of time when you experience symptoms (or need to take medication to treat symptoms) continuously’.

Participants received £10 upon completion of the survey. All subjects were free to withdraw from participation in this study at any time, and for any reason.

### Statistics

Statistical analyses were conducted in STATA 12 to compare baseline characteristics and exposures for mild disease to moderate/severe disease. For the purpose of analysis, participants with moderate/severe AR were defined as those who scored a rTNSS ≥8 out of 12, including a congestion score ≥2/3, when describing their ‘worst symptoms’. These rTNSS and nasal congestion score cut-offs were chosen in order to align with moderate/severe definitions from a recently conducted clinical trial [38]. Participants with mild disease were the remaining patients. The number of patients with mild and moderate/severe AR in both groups was very similar whether severity was classified according to rTNSS and congestion scores or according to the ARIA definition.

Student t tests and Wilcoxon rank-sum tests were used to compare continuous outcomes for the two SAR severities, for parametric and non-parametric data, respectively. Results are presented with means and standard deviations, unless significant skew was observed in the outcome, in which case medians are presented. Chi-squared tests and Fisher’s exact tests (where cell frequency was less than 5) were used to compare categorical outcomes to investigate differences between the two SAR severities and results presented as frequencies and percentages. Odds ratios were calculated for moderate/severe versus mild SAR for a given exposure with reference to no exposure. For all analyses p values <0.05 were judged to be statistically significant.

## Results

### Survey response

The survey was sent to 1300 potential participants. Data collection was stopped once 1000 patients completed the survey.

### Demographic and socioeconomic characteristics

One thousand SAR patients were recruited (mild: n = 254; moderate/severe: n = 746). The average age was 42.6 [standard deviation (SD) 12.1] years, with female gender and white ethnicity predominating (Table 1). Most participants were in full or part-time employment or self-employed (69.1 %), with over three quarters (76.9 %) educated to A-level standard (i.e. international baccalaureate level or above).

**Table 1:** Participant demographic and baseline data

	Allergic rhinitis severity		p value
	Mild (n = 254)	Moderate/severe (n = 746)	
Age, mean (sd)	44.1 (13.0)	42.1 (11.8)	0.0274
Gender, n (%) female	175 (68.9)	503 (67.4)	0.665
*Ethnicity, n (%)*			
White	226 (89.0)	666 (89.3)	0.894
Asian	16 (6.3)	41 (5.5)	0.633
Black	3 (1.2)	25 (3.4)	0.070
Mixed	5 (2.0)	5 (0.7)	0.072
No response	4 (1.6)	9 (1.2)	0.654
*Allergen sensitivity (self-reported)*			
Grass pollen	165 (65.0)	579 (77.6)	<0.001
Tree pollen	119 (46.9)	462 (61.9)	<0.001
Weed pollen	56 (22.0)	259 (34.7)	<0.001
Animals	57 (22.4)	231 (31.0)	<0.001
Mites	29 (11.4)	163 (21.8)	<0.001
Moulds	25 (9.8)	152 (20.4)	<0.001
Not sure	57 (22.4)	96 (12.9)	<0.001
Other	25 (9.8)	83 (11.1)	0.569
No. symptom episodes/year, median	6.0	8.0	0.025
*No. days/episode*			
Mean (SD)	9.8 (18.1)	12.5 (20.2)	0.0041
Median	4.0	5.0	0.013
Asthma diagnosis, n (%)	70 (30.4)	257 (35.8)	0.1368

SAR severity: participants with moderate/severe AR were defined as those who scored a rTNSS ≥8 out of 12, including a congestion score ≥2/3, when describing their ‘worst symptoms’. Participants with mild AR included all remaining patients

*SD* standard deviation

### Sensitization pattern

Grass and tree pollen were the most commonly reported sensitizing allergens, but indoor allergen (e.g. to animal dander, mites) and mould sensitization was also common. A high level of polysensitization was apparent particularly in the moderate/severe group (Table 1). Significantly (p < 0.001) more patients with moderate/severe disease were aware of their sensitizing allergen (Table 1).

### Episode pattern and duration

Patients with moderate/severe AR experienced significantly more symptomatic episodes/year than those with mild disease (median 8.0 vs 6.0; p = 0.025) with each of these episodes lasting significantly longer (12.5 vs 9.8 days; p = 0.0041; Table 1).

### Medication usage

Almost all patients reported taking medication to treat their rhinitis symptoms (90.6 and 96.2 % of patients with mild and moderate/severe AR, respectively). Oral H1-antihistamines were the medications most commonly reported, followed by INS (Table 2). Patients with moderate/severe AR were more likely to report nasal spray use (66.7 %) than those with mild disease [58.3 %; odds ratio (OR) 1.44; 95 % confidence interval (CI) 1.05–1.97; p = 0.0196]. One-third of patients in both groups used ocular medication (Table 2). Only 0.9 and 1.7 % of patients with mild or moderate/severe disease, respectively, reported use of injections (either immunotherapy or systemic corticosteroids) to treat their AR.

**Table 2:** Medication usage in mild and moderate/severe seasonal allergic rhinitis patients

	SAR severity			
	Mild (n = 254)	Moderate/severe (n = 746)	Odds ratio (95 % CI)	P value
Taking medication, n (%)	230 (90.6)	718 (96.2)	2.68 (1.45, 4.89)	0.0004
*Oral medications, n (%)*	*184 (80.0 %)*	*605 (84.3 %)*	*1.34 (0.89, 1.98)*	*0.1322*
Cetirizine	82 (44.6)	313 (51.7)	1.33 (0.94, 1.89)	0.0885
Loratadine	61 (33.2)	195 (32.2)	0.96 (0.67, 1.39)	0.8153
Chlorphenamine	61 (33.2)	178 (29.4)	0.84 (0.58, 1.22)	0.3349
Pseudoephedrine	14 (7.6)	92 (15.2)	2.18 (1.19, 4.25)	0.0081
Phenylephrine	7 (3.8)	33 (5.5)	1.46 (0.62, 3.97)	0.3716
Acrivastine	20 (10.9)	82 (13.6)	1.29 (0.75, 2.28)	0.3420
Levocetirizine	0 (0)	19 (3.1)	–	0.011
Fexofenadine	10 (5.4)	38 (6.3)	1.17 (0.56, 2.68)	0.6741
Desloratadine	3 (1.6)	24 (4.0)	2.49 (0.74, 13.06)	0.1651
Other	17 (9.2)	57 (9.4)	1.02 (0.57, 1.93)	0.9408
*Nasal sprays, n (%)*	*134 (58.3 %)*	*479 (66.7 %)*	*1.44 (1.05, 1.97)*	*0.0196*
Fluticasone propionate	96 (71.6)	338 (70.6)	0.95 (0.60, 1.47)	0.8083
Beclomethasone	33 (24.6)	110 (23.0)	0.91 (0.57, 1.48)	0.6875
Mometasone	4 (3.0)	31 (6.5)	2.25 (0.77, 8.92)	0.1241
Fluticasone furoate	4 (3.0)	12 (2.5)	0.89 (0.26, 3.84)	0.8401
Flunisolide	1 (0.8)	12 (2.5)	3.42 (0.50, 147.15)	0.2116
Budesonide	2 (1.5)	10 (2.1)	1.41 (0.29, 13.36)	0.6602
Ipratropium bromide	0 (0)	5 (1.0)	–	0.29
Other	18 (13.4)	48 (10.0)	0.72 (0.39, 1.36)	0.2600
Oxymetazoline	9 (6.7)	39 (8.1)	1.23 (0.57, 2.97)	0.5871
Azelastine	25 (18.7)	106 (22.1)	1.23 (0.75, 2.10)	0.3860
*Ocular medications, n (%)*	*72 (31.3 %)*	*275 (38.3 %)*	*1.36 (0.98, 1.90)*	*0.0552*
Sodium cromoglicate	14 (19.4)	82 (29.8)	1.76 (0.91, 3.61)	0.0798
Antazoline	12 (16.7)	50 (18.2)	1.11 (0.54, 2.44)	0.7651
Xylometazoline	9 (12.5)	36 (13.1)	1.05 (0.47, 2.62)	0.8943
Azelastine	3 (4.2)	13 (4.7)	1.14 (0.30, 6.41)	0.8400
Olopatadine	3 (4.2)	17 (6.2)	1.52 (0.42, 8.29)	0.5137
Lodoxamide trometamol	1 (1.4)	9 (3.3)	2.40 (0.32, 106.74)	0.3950
Other	33 (45.8)	98 (35.6)	0.65 (0.37, 1.15)	0.1121
*Co-medicating, n (%)*	*129 (56.1)*	*506 (70.5)*	*1.87 (1.36, 2.56)*	*0.0001*
Reported reason for co-medicating, n (%)				
More effective nasal treatment	55 (42.6)	295 (58.3)	1.88 (1.25, 2.84)	0.0014
More effective ocular treatment	54 (41.9)	209 (41.3)	0.98 (0.65, 1.48)	0.9089
Faster nasal response	22 (17.1)	116 (22.9)	1.45 (0.86, 2.52)	0.1490
Faster ocular	13 (10.1)	57 (11.3)	1.13 (0.59, 2.33)	0.7007
Other	18 (19.0)	48 (9.5)	0.65 (0.35, 1.23)	0.1378

SAR severity: paricipants with moderate/severe AR were defined as those who scored a rTNSS ≥8 out of 12, including a congestion score ≥2/3, when describing their ‘worst symptoms’. Participants with mild AR included all remaining patient

*SAR* seasonal allergic rhinitis, *CI* confidence interval

Most patients reported the use 2 or more AR medications (56.1 % of patients with mild AR and 70.5 % of patients with moderate/severe AR), but were nearly twice as likely to do so if they had moderate/severe disease (OR: 1.87; 95 % CI 1.36–2.56; p = 0.0001) (Table 2). The search for better nasal symptom relief, was the most common reason reported by patients for taking 2 or more AR medications. This was particularly evident in the moderate/severe group, where 58.3 % of patients cited the need for more effective nasal treatment as the reason for co-medicating compared to 42.6 % of those with mild AR (OR 1.88; 95 % CI 1.25–2.84; p = 0.0014) (Table 2). More effective ocular symptom relief was another important determinant governing co-prescribing behaviour, reported by over 40 % of patients in both groups (Table 2). This was in line with the proportion of patients who reported ocular medication use (mild: 31.3 %; moderate/severe: 38.3 %). The search for faster response also drove AR treatment choice, with almost 35 % of patients with moderate/severe AR citing this as their reason for co-medicating (Table 2).

### Symptom burden

**Fig. 1:**
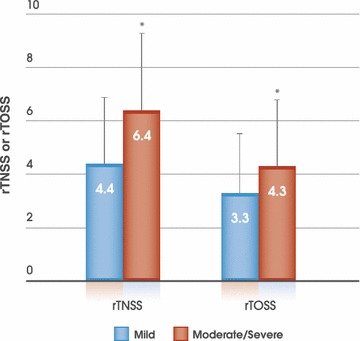
Nasal and ocular symptom burden reported by seasonal allergic rhinitis patients with mild (n = 254) or moderate/severe disease (n = 746) on the day of assessment. Over 90 % of these patients in both groups were taking AR medication (see Table 2). Data are presented as mean and standard deviation. rTNSS: reflective total nasal symptom score (max = 12); rTOSS: reflective total ocular symptom score (max = 9). *p < 0.0001 vs mild AR

The symptom burden reported by these patients was high, even though over 90 % of them were taking an AR medication. On the day of assessment, participants in both severity groups reported significant nasal and ocular symptoms. However, this burden (both nasal and ocular) was significantly higher in those with moderate/severe disease (Fig. 1). Patients with moderate/severe disease also reported a significantly (p < 0.0001) higher overall nasal symptom burden when symptoms were at their worst (10.0 [SD 1.5] vs 5.9 [SD 1.9]).

**Fig. 2:**
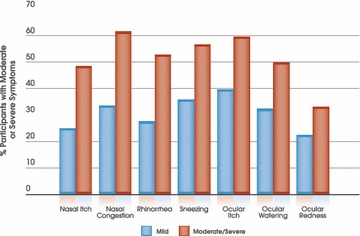
Proportion of patients with mild (n = 254) or moderate severe AR (n = 746) scoring a ‘2’ (moderate) or ‘3’ (severe) for individual nasal and ocular symptom scores on the day of assessment. Over 90 % of these patients in both groups were taking AR medication (see Table 2). Significance values for mild vs moderate/severe groups are given for each level of symptom severity in Table 3

On the day of assessment (June–July 2011), many patients were experiencing ‘moderate’ or ‘severe’ nasal itch, congestion, rhinorrhea and sneezing as well as ocular itch, watering and redness, despite treatment, with significantly more patients with moderate/severe AR experiencing greater symptom severity for each nasal and ocular symptom (Table 3; Fig. 2). Congestion appeared to be the most bothersome nasal symptom; with 61.5 % of participants with moderate/severe AR rating its severity as ‘moderate’ or ‘severe’ on the day of assessment compared to 33.5 % of those with mild disease (Fig. 2). Ocular itch was the most bothersome ocular symptom; 59.4 % patients with moderate/severe AR rated its severity as ‘moderate’ or ‘severe’ on the day of assessment compared to 39.7 % of those with mild AR (Fig. 2).

**Table 3:** Nasal and ocular symptom burden of patients with mild and moderate/severe AR on the day of assessment

Symptom	Symptom severity	SAR severity		P value
		Mild (n = 254)	Moderate/severe (n = 746)	
*Nasal symptoms of the rTNSS*				
Nasal itch, n (%)	None	63 (24.8)	87 (11.7)	<0.001
	Mild	128 (50.4)	298 (39.9)	0.004
	Moderate	54 (21.3)	283 (37.9)	<0.001
	Severe	9 (3.5)	78 (10.5)	0.001
Nasal congestion, n (%)	None	67 (26.4)	61 (8.2)	<0.001
	Mild	102 (40.2)	226 (30.3)	0.004
	Moderate	67 (26.4)	312 (41.8)	<0.001
	Severe	18 (7.1)	147 (19.7)	<0.001
Rhinorrhea, n (%)	None	82 (32.3)	111 (14.9)	<0.001
	Mild	102 (40.2)	241 (32.3)	0.023
	Moderate	56 (22.0)	279 (37.4)	<0.001
	Severe	14 (5.5)	115 (15.4)	<0.001
Sneezing, n (%)	None	55 (21.7)	68 (9.1)	<0.001
	Mild	108 (42.5)	256 (34.3)	0.019
	Moderate	75 (29.5)	281 (37.7)	0.019
	Severe	16 (6.3)	141 (18.9)	<0.001
*Ocular symptoms of the rTOSS*				
Ocular itch, n (%)	None	51 (20.1)	97 (13.0)	0.006
	Mild	102 (40.2)	206 (27.6)	<0.001
	Moderate	74 (29.1)	276 (37.0)	0.023
	Severe	27 (10.6)	167 (22.4)	<0.001
Ocular watering, n (%)	None	70 (27.6)	154 (20.6)	0.022
	Mild	102 (40.2)	220 (29.5)	0.002
	Moderate	64 (25.2)	239 (32.0)	0.040
	Severe	18 (7.1)	133 (17.8)	<0.001
Ocular redness, n (%)	None	91 (35.8)	183 (24.5)	<0.001
	Mild	106 (41.7)	316 (42.4)	0.861
	Moderate	51 (20.1)	209 (28.0)	0.013
	Severe	6 (2.4)	38 (5.1)	0.067

SAR severity: paricipants with moderate/severe AR were defined as those who scored a rTNSS ≥8 out of 12, including a congestion score ≥2/3, when describing their ‘worst symptoms’. Participants with mild AR included all remaining patients

Symptom severity: Assessed by individual symptom scores of the rTNSS and rTOSS; 0 = none, 1 = mild, 2 = moderate, 3 = severe

*SAR* seasonal allergic rhinitis, *rTNSS* reflective total nasal symptom score, *rTOSS* reflective total ocular symptom score

### Health care visits

Participants with moderate/severe AR reported visiting their GP for their SAR more frequently than those with mild AR (1.61 vs 1.19 times/year; OR 1.49; 95 % CI 1.11–2.01; p = 0.0061). In both groups, dissatisfaction with treatment was a primary reason for the visit; 28 % of visits for patients with mild AR versus 35 % of visits for those with moderate/severe disease, with patients with moderate/severe AR being significantly more likely to report treatment dissatisfaction than those in the mild group (OR 1.49; 95 % CI 1.06–2.13; p = 0.0194).

### Impact on asthma

Many AR participants reported co-morbid asthma; 30.4 and 35.8 % of participants with mild and moderate/severe AR, respectively, and reported modifying their asthma medication (both reliever and controller) if they failed to take their AR medication. Patients with moderate/severe AR were twice as likely to describe this behaviour. For asthma reliever medication, 45.7 % of patients with mild AR with co-morbid asthma (n = 70) reported increased use compared to 53.7 % of patients with moderate/severe AR (n = 257) (OR 1.93; 95 % CI 1.01–3.68; p = 0.0303). Similarly, 15.7 % of patients with mild AR with co-morbid asthma reported the need to increase their controller medication if they failed to take their AR medication, rising to 19.5 % of patients in the moderate severe group (OR 2.04; 95 % CI 0.86–5.03; p = 0.0781).

### Absenteeism and presenteeism

Patients with moderate/severe AR reported absenteeism from work due to their SAR on 4.1 (SD 16.4) days/year compared to 2.5 (SD 7.7) days/year for patients in the mild group (OR: 1.34; 95 % CI: 0.87-2.11; p = 0.1708). This was significantly more likely for patients with moderate/severe AR who reported 37.7 (SD 53.0) days/year when their productivity was affected by their SAR symptoms, almost double that noted by patients with mild disease (21.0 days [SD 29.9]; OR: 1.71; 95 % CI: 1.15-2.54; p = 0.0048).

**Fig. 3:**
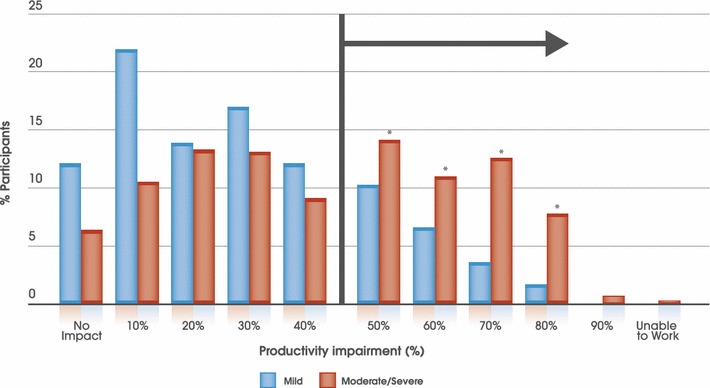
Presenteeism due to SAR reported by patients with mild disease (n = 164) and those with moderate/severe disease (n = 521). *p ≤ 0.0093 vs mild AR. Patients with moderate/severe AR significantly (OR 3.52; CI 2.10–6.13; p < 0.0001) more likely than those with mild AR to have a >50 % impairment in their work productivity due to their SAR symptoms

Participants with mild AR did report some negative impact on their productivity, clustered predominantly at the lower impact end of the productivity scale (i.e. < 50 % impact). The negative impact on participant-reported work productivity due to SAR symptoms was much more apparent for those with moderate/severe disease. These patients were almost 4 times more likely to experience > 50 % negative impact on their work productivity than those with mild disease (32.8 % vs 12.2 %; OR: 3.52; 95 % CI: 2.10-6.13; p < 0.0001) (Fig. 3).

## Discussion

This study provides a comprehensive view of the AR burden and unmet need in the UK. A complete dataset has been collected from a medically- diagnosed, symptomatic, SAR patient population (of similar disease severity to those included in a recent SAR study) [39] including information on SAR episode pattern and duration, medication/co-medication usage, reasons for co-medication, residual symptoms on treatment, number of healthcare visits, absenteeism and productivity loss in patietns with mild and moderate/severe AR. It, therefore, represents a complete assessment of AR burden and unmet need in a single survey.

This was a relatively large survey, including 1000 AR patients with wide representation of age, educational level and employment status. Survey content was broad and informed by several world-renowned experts in the field of AR. As this was an online survey, there was no interviewer bias. Responders were free to answer the questions in a time convenient to them and at their own pace. Patients were initially screened for severity using the Allergic Rhinitis and its Impact on Asthma (ARIA) severity classification system yielding 200 patients with mild AR and 800 patients with moderate/severe AR to ensure adequate representation of patients with moderate/severe AR in the survey (i.e. patients most likely to visit their healthcare provider). However, to align with moderate/severe definition commonly employed in AR clinical trials, severity was also classified using rTNSS and congestion score cut offs for the purpose of data analysis. Very similar numbers were reported using this method of categorization; 254 and 746 for patients with mild and moderate/severe AR, respectively. This confirms the robustness of the ARIA severity definition as a quick, simple and accurate method of severity categorization, and also that the moderate/severe definition used in the present analysis largely conforms to ARIA.

Although the data relates to the UK in terms of allergen exposure, as well as treatment and referral patterns, the results also have a broader relevance for clinical trial design in general. For example, knowledge of the duration of a typical mild and moderate/severe SAR symptom episode could inform trial duration decisions and also encourage contextualization of efficacy endpoints with a temporal focus. A potential limitation of this survey was that patients were recruited from a patient panel. These panels include a varied and heterogeneous patient population. Panel patients are not subjected to stringent inclusion/exclusion criteria and have a relaxed ecology of care making the information they provide more indicative of the real world. Conversely, AR patients recruited into randomized controlled trials (RCTs) are poorly representative of those seen in primary care [40]. In the present study AR was classified according to time of year when symptoms appeared (i.e. SAR) rather than the ARIA classification based on symptom longevity (i.e. intermittent/persistent). These classifications are not interchangeable [1], and whilst the SAR/PAR classification is still widely used in primary care, the newer (and more therapeutically relevant) ARIA classification system should be promoted at both the patient and physician level. By design, most patients included in the survey had moderate/severe disease and so represent the type of patients who present to physicians [2, 4, 17, 23]. Also, patients were included in this survey based on a reported medical diagnosis of SAR, rather than a medically-confirmed diagnosis. No data were collected on irritant exposure or smoking history. It would have been interesting to examine their impact on symptom burden and therapeutic response. As with all surveys of this nature there was a reliance on patient recall. Variability was noted for some responses as evidenced by large standard deviations around the mean. Where this occurred, median values were used.

The survey found that patients experienced several symptomatic bursts throughout the year, each lasting for some days, with participants with moderate/severe AR reporting significantly greater symptom episode frequency and duration than their milder counterparts. There was a clear symptom burden shift from patients with mild to those with moderate/severe AR, the latter, more likely to report more and longer episodes/year. These facts were previously unrecognised. The symptom burden shift provides evidence of the quality of the survey data and its sensitivity to discriminate according to disease symptom severity. Knowledge of duration and frequency of AR symptom episodes is important to know when assessing the symptomatic and economic burden of AR, and when considering treatment choice. It indicates that rapid relief of symptoms is important to control the disease.

The extent to which patients co-medicate is underestimated by physicians and payers alike, since over the counter medications are frequently added to prescription medications. This finding has also been observed in Spain and France [3, 26, 27]. The majority of participants who took part in this survey reported using 2 or more AR medications (most commonly an INS plus an oral H1-antihistamine) in an attempt to achieve better and faster nasal and ocular symptom relief. This was true for both the participants with moderate/severe disease (70.5 %) and those with mild AR (56.1 %), although significantly more likely in those with more severe disease. Therefore, the direct cost of AR may be higher than previously thought, as patients supplement with multiple treatments, driven by their search for better efficacy. This search for a faster and more effective nasal therapy was more in evidence as a driver for those patients with moderate/severe AR emphasizing the higher symptom burden of this group, not only in terms of symptom severity, but also in terms of episode frequency and duration. The fact that over half of patients with mild AR co-medicated was an unexpected finding. This result showed that monotherapy provides insufficient symptom relief for a substantial proportion of patients with mild AR too, suggesting that they may underestimate the true severity of their disease and/or rely on over-the-counter AR medications, being resistant to attending their physician in order to receive a more effective treatment option, or indeed a more accurate severity diagnosis. Others have confirmed that co-mediation prescribing behaviour has been steadily rising in the UK in the last 2 decades; dual therapy has doubled since 1992, whilst use of triple therapy has increased eight-fold [41].

However, co-medication does not appear to provide the symptom relief, which AR patients seek. Logically, one would assume that use of several medications from different classes would provide improved pathologic coverage leading to better symptom control. But, this does not appear to be the case. The present survey results confirm the results obtained in randomized clinical trials [29, 30]. Both patients with mild and moderate/severe AR included in this survey remained symptomatic, with those with more severe disease more likely to be so, even though > 90 % of patients were on AR treatment, and many were co-medicating. In other words, patients’ symptoms were still of moderate severity, on average, despite treatment. Nasal congestion and ocular itching remained problematic for 60 % of patients with moderate/severe disease and were difficult to control with mono or multiple therapies. A similar pattern of mono- and multiple-therapy insufficiency has also been observed in other countries [4]. There is, therefore, a clear need for a faster and more effective AR treatment option with wide symptomatic and pathologic coverage, which provides more complete and rapid symptom control. MP29-02, comprising azelastine hydrochloride, fluticasone propionate and a novel formulation in a single spray, is the newest addition to the AR treatment arsenal and is promising in this regard [39, 42]. Allergen-specific immunotherapy should be strongly considered for patients who fail to respond to symptomatic therapy, particularly for those patients for whom symptoms are predominantly caused by one allergen [43], and may significantly reduce the burden of AR in these patients.

This survey also serves to highlight the large indirect burden of AR in the UK; the hidden costs associated with this disease are substantial. Many patients with AR also have asthma, with failure to control one having a detrimental effect on control of the other [1]. In the present survey, asthma medication usage (both reliever and controller) was likely to be increased by participants if they failed to use their AR medication, and more likely to occur in those with moderate/severe AR. Other indirect costs reported included absenteeism and presenteeism. On average, patients with moderate/severe AR reported 4 days/year absent from work due to their SAR. Assuming an average cost of £71 for each lost day [44], this amounts to £1.14 billon/year in the UK alone. This figure does not take presenteeism into consideration, which was reportedly negatively impacted on 38 days/year and carries a substantial indirect cost [19].

Knowledge of AR symptom patterns is vital when considering relevancy of clinical trial data and appropriateness of clinical trial design. Patients with intermittent AR (as categorized by ARIA) experience symptoms for <4 days/week or for less than 4 consecutive weeks [1]. Based on the results presented here, we now have corresponding information for SAR (i.e. average symptom episode lasts 9.8 days for mild SAR and 12.5 days for moderate/severe SAR). Therefore, SAR trials of 14 days duration are sufficiently long to assess the clinical efficacy of medications in most patients; since this timeframe spans a single episode, and thus reflects the real-world situation. Additionally, any improvements afforded by AR medications in patients with moderate/severe AR should now be contextualized and assessed for clinical relevancy within a 12.5 day time frame. It is also clear that direct head-to-head trials of active comparators are needed, not simply comparisons versus placebo, since the vast majority of patients with moderate/severe AR are treated, and most are co-medicating. Therefore, studies versus placebo only, in those patients with moderate/severe disease are not clinically-relevant, may provide a distorted view of the effectiveness of active comparators, and are likely to increase the number of insufficiently effective drugs registered, failing to meet patient expectations of treatment. The results of our study support the request of ARIA to conduct clinical trials against gold standard therapy in order to show clinically relevant improvements that will lower the burden of AR and improve its management. A recently published state of the art analysis of a new AR therapy, is an important first step in this direction [39]; (1) patients included in the trial had moderate/severe disease, representing the type of patient commonly seen in practice, (2) first-line AR medications were used as active comparators (in addition to placebo), (3) results were contextualised within a typical symptom episode window and (4) data were analysed to show not only superior efficacy to established first line therapies but also a faster response, which is what patients want [33, 45].

The impact of patients’ attitudes on their AR health outcomes and their decision processes when considering which AR medication to take are interesting avenues for additional research. More information on patient knowledge (both about the disease and available treatments) as well as incidence of co-morbidities (e.g. food allergy, asthma, atopic dermatitis) would also provide a more global look at burden of care. Finally, patients should be empowered to take responsibility for their own AR control, encouraged to improve their disease awareness and knowledge of AR therapeutic options and improve concordance with their treatment regimen. In this regard, the importance of a common AR control concept and language (for both patients and physicians) has been recognized [46]. MACVIA ARIA has recently launched an app, called Allergy Diary, which uses a simple visual analogue scale (VAS) to assess control and will use this same VAS in an app for health care providers (called Allergy Diary Companion) and in the updated guideline to guide AR treatment decisions [46].

## Conclusions

This cross-sectional online questionnaire-based study represents a comprehensive assessment of the burden and unmet need of AR in the UK in a large patient population. Knowledge of the results of study should be used to inform clinical trial design and relevancy of clinical findings, and to assess the potential impact of AR treatments on the true burden and unmet need in this highly prevalent condition.

## Additional file

10.1186/s13601-015-0083-6 Socio-demographic/healthcare utilisation questions.

## Abbreviations

AR: allergic rhinitis
ARIA: allergic rhinitis in asthma
CI: confidence interval
INS: intranasal corticosteroid
OR: odds ratio
RCT: randomized controlled trial
rTNSS: reflective total nasal symptom score
rTOSS: reflective total ocular symptom score
SAR: seasonal allergic rhinitis
SCUAD: severe chronic upper airway disease
SD: standard deviation
WPAI: work productivity and activity impairment
