# Supplementary material for: The hidden burden of adult allergic rhinitis: UK healthcare resource utilisation survey
Source: Clin Transl Allergy. 2015 Nov 18;5:39. doi: 10.1186/s13601-015-0083-6 (PMC4650835; doi:10.1186/s13601-015-0083-6)
Supplement: Supplementary file 1 — 10.1186/s13601-015-0083-6 Socio-demographic/healthcare utilisation questions. [file 13601_2015_83_MOESM1_ESM.doc]

**Additional file**

Socio-demographic/healthcare utilisation questions

1. What is your age?

_________________________________

1. Gender:
   1. Male
   2. Female
2. How would you describe your ethnicity? (tick one option only)
   1. White
   2. Black
   3. Asian
   4. Mixed Ancestry
   5. *Prefer not to answer*
3. What is your **main** occupation? (tick one option only)
   1. Employed full time
   2. Employed part time
   3. Self employed
   4. Home maker
   5. Student
   6. Retired, including medically
   7. Unemployed
   8. Other: __________________________
   9. *Prefer not to answer*
4. What is your **personal** income per year? (tick one option only)
   1. Less than £10,000
   2. £11-20,000
   3. £21-30,000
   4. £31-50,000
   5. £51-75,000
   6. £76 – £100,000
   7. More than £100,000
   8. *Prefer not to answer*
5. What is your **highest level** of education? (tick one option only)
   1. No formal qualifications
   2. GCSEs or equivalent
   3. A Level of equivalent
   4. Technical/ vocational qualifications
   5. University level
   6. Other: ____________________________
   7. *Prefer not to answer*
6. What do you think are the **main causes** of your hay fever/seasonal allergic rhinitis symptoms? (tick all that apply)
   1. Tree pollen
   2. Grass pollen
   3. Weed pollen
   4. Moulds
   5. *Not sure*
   6. Other:
      - 1. ____________________________
        2. ____________________________
        3. ____________________________
        4. ____________________________
7. During which months of the year do you experience hay fever/seasonal allergic rhinitis symptoms? (tick all that apply)
   1. January
      1. Approximately **how many** **days** do you experience symptoms in this month? *(repeated for each selected month)*
         1. 1-31 days (drop down list with correct number of days / month)
   2. February
   3. March
   4. April
   5. May
   6. June
   7. July
   8. August
   9. September
   10. October
   11. November
   12. December
8. **Within a year**, approximately how many **separate symptom episodes**  of hay fever/ seasonal allergic rhinitis do you experience (an episode is a period of time when you experience symptoms continuously)?

______________________ episodes

1. Approximately **how many days** **does each symptom episode** last?

______________________ days

1. Do you take any medication to treat your hay fever/seasonal allergic rhinitis?
   1. Yes
   2. No *(go to question Q19)*
2. Do you use **fluticasone nasal spray (also known as** **Flonase, Flixonase, Nasofan, Pirinase or Avamys**) to treat your hay fever / seasonal allergic rhinitis symptoms?
   1. Yes
   2. No *(go to question Q13)*

*If yes*

- 1. How do you **usually** get this medication?(tick one option only)
     1. On prescription
     2. I buy it from a pharmacy/chemist **without** a prescription
  2. Approximately **how many days** do you take this medication for **during a symptom episode**? ________ days
  3. Do you **re-use** this medication **across symptom episodes** (e.g. when you have medication left after one symptom episode do you use this left over medication when your symptoms return)? Y/N
  4. **How many symptom episodes** would a **30-day treatment usually** cover?

_________ episodes

1. Do you use **azelastine nasal spray (also known as Astelin or Rhinolast)** to treat your hay fever/ seasonal allergic rhinitis symptoms?
   1. Yes
   2. No *(go to question Q14)*

***If yes***

- 1. How do you **usually** get this medication?(tick one option only)
     1. On prescription
     2. I buy it from a pharmacy/chemist **without** a prescription
  2. Approximately **how many days** do you take this medication for **during a symptom episode**? ________ days
  3. Do you **re-use** this medication **across symptom episodes** (e.g. when you have medication left after one symptom episode do you use this left over medication when your symptoms return)? Y/N
  4. **How many symptom episodes** would a **30-day treatment usually** cover?
     1. _________ episodes

1. Do you use any **other nasal sprays** to treat your hay fever / seasonal allergic rhinitis?
   1. Yes
   2. No *(go to question Q15)*

***If yes***

1. Which **other nasal sprays** do you use? **(*in survey there will be more than two boxes*)**
   - 1. Name of nasal spray 1 (please type name):  *___________ (name of nasal spray will be retrieved from database)*
     2. Name of nasal spray 2 (please type name): ___________ (*name of nasal spray will be retrieved from database)*
     3. Unsure of name of nasal spray

**[*Participants answer all of the following questions for each nasal spray***]

1. How do you **usually** get this medication?(tick one option only)
   - 1. On prescription
     2. I buy it from a pharmacy/chemist **without** a prescription
2. Approximately **how many days** do you take this medication for **during a symptom episode**? ________ days
3. Do you **re-use** this medication **across symptom episodes** (eg. when you have medication left after one symptom episode do you use this left over medication when your symptoms return)? Y/N
4. **How many symptom episodes** would a **30-day treatment usually** cover?

_________ episodes

1. Do you use any **oral medications** to treat your hay fever / seasonal allergic rhinitis (e.g. tablets, capsules or liquid?
2. Yes
3. No *(go to question Q16)*

***If yes***

1. Which **oral medications** do you use?
2. Name of oral medication 1 (please type name):  *___________ (name of oral medication will be retrieved from database)*
3. Name of oral medication 2 (please type name): ___________ (*name of oral medication will be retrieved from database*)
4. Unsure of name of oral medication

**[*Participants answer all of the following questions for each oral medication***]

1. What type of medication is this?(tick one option only)
   - 1. Tablet
     2. Capsule
     3. Liquid
2. How do you **usually** get this medication?(tick one option only)
   - 1. On prescription
     2. I buy it from a pharmacy/chemist **without** a prescription
3. Approximately **how many days** do you take this medication for **during a symptom episode**? ________ days
4. Do you **re-use** this medication **across symptom episodes** (e.g. when you have medication left after one symptom episode do you use this left over medication when your symptoms return)? Y/N
5. **How many symptom episodes** would a **30-day** treatment usually cover?

_________ episodes

1. Do you use any **eye drops** to treat your seasonal hay fever /seasonal allergic rhinitis?
   1. Yes
   2. No *(go to question Q17)*

***If yes***

1. Which **eye drops** do you use?
2. Name of eye drops 1 (please type name):  *___________ (name of eye drops will be retrieved from database)*
3. Name of eye drops 2 (please type name): ___________ (*name of eye drops will be retrieved from database)*
4. Unsure of name of eye drops

**[*Participants answer all of the following questions for each ocular medication***]

1. How do you **usually** get this medication?(tick one option only)
   - 1. On prescription
     2. I buy it from a pharmacy/chemist **without** a prescription
2. Approximately **how many days** do you take this medication for **during a symptom episode**? ________ days
3. Do you **re-use** this medication **across symptom episodes** (e.g. when you have medication left after one symptom episode do you use this left over medication when your symptoms return)? Y/N
4. **How many symptom episodes** would a **30-day treatment usually** cover? _________ episodes
5. Do you have any **injections** to treat your hay fever / seasonal allergic rhinitis?
   1. Yes
   2. No *(go to question Q18)*

***If yes***

1. Which injections do you have?
2. Name of injection 1 (please type name):  *___________ (name of injection will be retrieved from database)*
3. Unsure of name of injection
4. How do you usually get this medication?
   - 1. On prescription from my GP
     2. On prescription covered by health insurance
     3. I pay for my injection out of my own pocket
5. **Why** do you take **more than one medication** to treat your hay fever/seasonal allergic rhinitis? (tick all that apply)
   1. Not applicable, I only use 1 medication
   2. One treatment does not treat all of my nasal symptoms effectively
   3. I need additional treatment for my eye symptoms
   4. One treatment does not treat my symptoms fast enough
   5. Other _______________
6. Approximately **how many** **visits in a year** do you make to your GP/nurse because of hay fever/ seasonal allergic rhinitis, i.e. a face-to-face consultation?
   1. GP _______ visits
   2. Nurse _______ visits
7. **How many of these visits** are made in order to **discuss dissatisfaction** with your seasonal allergic rhinitis/hay fever medication?
   1. GP _______ visits
   2. Nurse ________visits
8. Do you have a **clinical diagnosis** of asthma?
   1. Yes
   2. No *(skip to question 23)*

***If yes:***

1. If you **don’t** take your hay fever/seasonal allergic rhinitis medication when symptomatic, how is your asthma treatment affected? (tick one option only)
   1. Not affected at all
   2. I need to increase my reliever medication (normally blue in colour)
   3. I need to increase my preventer medication (usually brown, purple or red, in colour)
   4. I need to take additional steroid tablets
2. Are you in **paid** employment?
   1. Yes
   2. No *(skip to question 27)*

***If yes:***

1. How many **days off work**, due to hay fever/seasonal allergic rhinitis symptoms, would you typically take **during a year**? ________ days
2. How many **days** would your symptoms typically **affect your productivity** while at work, **during a year**? *(E.g. feeling limited in the amount of work you could do, accomplishing less than you would like, or not being able to do your work as carefully as usual because of your hay fever/ allergic rhinitis symptoms). ______* days
3. How would you **rate the impact** of hay fever/ allergic rhinitis on your **productivity**?

| No impact |  |  |  |  |  |  |  |  |  |  |  | Completely prevents me from working |
| --- | --- | --- | --- | --- | --- | --- | --- | --- | --- | --- | --- | --- |
|  | 0% | 10% | 20  % | 30% | 40% | 50% | 60% | 70% | 80% | 90% | 100% |  |
|  |  |  |  |  |  |  |  |  |  |  |  |  |

1. If a new, **fast acting** **nasal spray** was available which improves your symptoms **days earlier** than currently available therapies and has a **sustained effect** on both your **eye and nasal symptoms**, how much would you be willing to pay for this medication, **per month**?

£_______ per month
